# A Field Programmable Gate Array-Based Reconfigurable Smart-Sensor Network for Wireless Monitoring of New Generation Computer Numerically Controlled Machines

**DOI:** 10.3390/s100807263

**Published:** 2010-08-03

**Authors:** Sandra Veronica Moreno-Tapia, Luis Alberto Vera-Salas, Roque Alfredo Osornio-Rios, Aurelio Dominguez-Gonzalez, Ion Stiharu, Rene de Jesus Romero-Troncoso

**Affiliations:** 1 HSPdigital—CA Mecatronica, Facultad de Ingenieria, Campus San Juan del Rio, Universidad Autonoma de Queretaro, Rio Moctezuma 249, 76807 San Juan del Rio, Qro., Mexico; E-Mails: svmoreno@hspdigital.org (S.V.M.-T.); lavera@hspdigital.org (L.A.V.-S.); raosornio@hspdigital.org (R.A.O.-R.); auredgz@uaq.mx (A.D.-G.); 2 Department of Mechanical and Industrial Engineering, Concordia University, Montreal, Quebec H3G 1M8, Canada; E-Mail: istih@vax2.concordia.ca (I.S.); 3 HSPdigital—CA Telematica, DICIS, Universidad de Guanajuato, Carr. Salamanca-Valle km 3.5+1.8, Palo Blanco, 36885 Salamanca, Gto., Mexico

**Keywords:** smart sensors, wireless sensor network, FPGA, CNC monitoring

## Abstract

Computer numerically controlled (CNC) machines have evolved to adapt to increasing technological and industrial requirements. To cover these needs, new generation machines have to perform monitoring strategies by incorporating multiple sensors. Since in most of applications the online Processing of the variables is essential, the use of smart sensors is necessary. The contribution of this work is the development of a wireless network platform of reconfigurable smart sensors for CNC machine applications complying with the measurement requirements of new generation CNC machines. Four different smart sensors are put under test in the network and their corresponding signal processing techniques are implemented in a Field Programmable Gate Array (FPGA)-based sensor node.

## 1. Introduction

Since their inception, computer numerically controlled (CNC) machines have evolved to adapt to changing technological and industrial requirements. The predominant factor which drove the initial development of CNC machines was the need for increased productivity. The necessity of machining ever smaller components and to comply with the tougher standards that limit the number of defects in these components, requires CNC machines to not only increase their precision, speed and accuracy but also to maintain a high productivity. To cover these needs, new generation machines have to perform monitoring strategies to allow autonomous self-optimization that can be done through the integration of control-process strategies into the machine tool control architecture [1]. These capabilities are achieved by incorporating multiple sensors [2] for online measurement and processing that allow remote monitoring and intelligent maintenance for production maximization [3]. The installation of these sensors should not affect the machining process [4], therefore, the incorporation and use of a wireless sensor network is desirable.

In order to provide some of the desired features and functionalities, new generation CNC machines require the measurement of different variables that are useful for obtaining, in a direct or indirect form, the required information for diagnosing the machine’s operational condition and improving the machining process [1]. The most common-measured variables on a CNC machine include, among others, vibration, current, force and acoustic emissions. Different methodologies have been proposed to obtain different parameters or features of the process, as well as the tool and the machine conditions in general. Some early works that use the previously mentioned variables in their methodologies are Albarbar, *et al.* [5], Wright, *et al*. [6], Malekian *et al.* [7], Rangel-Magdaleno, *et al.* [8], Vela-Martinez *et al*. [9] that use vibrations for monitoring the electromechanical condition on the CNC machine, Romero-Troncoso *et al*. [10], Franco-Gasca *et al*. [11], Li *et al*. [12] that use current for detecting the cutting force and the tool wear, Huang *et al*. [13], Ertnuc and Oysu [14], Shao *et al*. [15] that use force measurements for monitoring the cutting force, Alonso and Salgado [4], Salgado and Alonso [16] use acoustic emissions for tool-wear monitoring. Likewise, there are other works related to the dynamic parameters, such as Morales-Velazquez *et al*. [17] and Petrella and Tursini [18]. By utilizing the dynamic motion parameters, it is possible to optimize the piece machining, minimize the tool wear, and increase the precision of the positioning platforms for micro-machining applications [7], where different techniques have been studied, such as those using interferometry, or those using position sensing detectors (PSD) in order to validate the resolution and compensate for errors [19,20]. Since in most of the applications the online processing of the variables is essential, the use of smart sensors is necessary [21]. Smart sensors have been developed and used for several applications in different works, as in those of Wang and Jianu [22], Rangel-Magdaleno, *et al.* [8], Petrella and Tursini [18], Ramamurthy *et al*. [23] and Son *et al*. [24]. However, new generation CNC machines require the measurement of different variables at the same time; therefore, a wireless network is necessary for solving the necessity of incorporating multiple sensors with specific processing capabilities.

Some sensor networks based on different wireless technologies have been developed, as in the case of Salvadori *et al*. [25], who proposed a system that uses two hardware topologies responsible for signal acquisition, processing, and transmission; one is based on an intelligent sensor module, and the other is a module for remote data acquisition. Wireless communication is accomplished through a transceiver using Gaussian Frequency-Shift Keying (GFSK) modulation in the 2.4 GHz band with a transmission rate of 1 Mbps, but it uses a custom-made protocol. Son *et al.* [24] proposed a wireless smart sensor network using the WiFi protocol which is based on the IEEE 802.11b standard, in which the communication is done through a commercial module that is used as a wireless bridge for the transmission of sensor data. On the other hand, Ramamurthy *et al*. [23] present a generic platform using WiFi and Bluetooth wireless protocols; the smart sensor node is composed of three layers in order to make the system more flexible and portable, yet, the resulting size makes it impossible to use the system in applications where the sensor has to be mounted in a limited area. Although the necessity of measuring a large number of variables in CNC machines is latent, most of the developed works have the limitation of incorporating multiple sensors on the machines; others do not have online processing capacity. Some smart sensors that allow this capacity have been developed, but they focus on the processing of a single variable. Other works that propose wireless sensor networks meet the bandwidth requirements, but with high power consumption, and because of the protocol used, the number of nodes can be insufficient for some CNC machine applications.

The contribution of this work is the development of a wireless network platform of smart sensors for CNC machine applications based on the IEEE 802.15.4 standard to comply with the measurement requirements of new generation machines, which are low power consumption and high scalability to allow the incorporation of a large number of network nodes. The sensor node prototype has a small size and low cost, and it consists of two parts: the wireless protocol and hardware signal processing (HSP). Both parts require high processing capabilities, so an FPGA (Field Programmable Gate Array) implementation provides the characteristic of reconfigurability to the sensor node allowing it to be configured in order to carry out a specific task, or reconfigured on-demand to carry out other tasks. In the case of the proposed network, this capability is available for each smart sensor, allowing multiple-signal processing in parallel, and making it suitable for online applications. In this work, four different smart sensors are put under test in the network and their corresponding signal processing techniques are implemented in the FPGA sensor node: (1) accelerometer-based vibrations monitoring, (2) sensorless jerk monitoring using an adaptive antisymmetric high-order FIR (finite impulse response) filter, 3) FPGA-based fused smart-sensor for tool-wear area quantitative estimation in CNC machine inserts, and 4) PSD-based micropositioning measurement.

## 2. Background

### 2.1. Communication Protocols

For the development of a wireless sensor network, the evaluation of different communication technologies is necessary. Some factors that should be considered are: bandwidth, transmission rate, total range, scalability and power consumption. The IEEE 802.11g standard has a maximum transmission rate of 54 Mbps, a maximum range of 150 m and defines a maximum number of 32 nodes for a local area network (LAN); it requires additional encoding, which results in an increase of the power consumption [26]. Another standard for a personal area network (PAN) is the IEEE 802.15.1 (Bluetooth); this standard has a transmission rate of 1 Mbps, a maximum range of 10 m and allows the integration of a network with a master device and up to seven slave devices that can only communicate with their master [27]. The IEEE 802.15.4 (ZigBee) standard has a transmission rate of 250 kbps and a maximum range of 1,000 m in its most recent versions. This standard works in three different frequency bands and could contain up to 65,536 nodes on a network and has low energy consumption [28]. Table 1 shows a comparative between standards as discussed above.

A sensor network for monitoring and failure-detection applications in a manufacturing cell with new generation machine tools requires a large amount of nodes because different variables are usually measured; the IEEE 802.15.4 standard allows the integration of 65,535 devices, which is considerably higher compared to most standards usually used for wireless sensor networks, in addition to other benefits such as its transmission range, latency and low power consumption, this standard is considered as the optimum for this application.

### 2.2. Measurement of Variables in Machine Tools

Different techniques have been studied in relation to machine-tool condition monitoring and process optimization, using various physical variables for analysis such as acceleration current and dynamic movement by obtaining variables such as position, velocity and mainly jerk. Similarly, studies related to micro-machining have been done, obtaining position measurements in the order of microns.

Vibration measurements are essential for detecting and diagnosing any deviations from normal operation conditions in machines [5]. When a machining process exceeds the desired limits, it may result in excessive vibration, poor quality of the end-piece finishing and permanent damage to the machine.

Cutting tools along with raw material represent the highest cost of the production process in a manufacturing industry [11]. Among several techniques to detect the cutting force during a manufacturing process, current monitoring is the cheapest method because it is based on measuring the control current in the servo driver and then extracts the cutting force component [10]. During a machining process, the motor current is directly related to the tool condition, where power consumption will be higher for a worn tool than for a sharp one in the same process [11]. In the case of wear monitoring, the variables that have been used include force, vibration, acoustic emission, cutting temperature and current [4].

Dynamic parameters are related as successive derivations by theoretically differentiating position signals, to obtain speed, acceleration and jerk [17]. Jerk monitoring, defined as the first derivative of acceleration, has become a major issue in CNC machines; for this reason, different works have treated the estimation of this parameter, either from encoder counts or from accelerometer measurements [8]. Particularly, jerk is related to machine wear, amplifier saturation and other undesired electromechanical conditions [17].

The miniaturization of components has become an important part of modern technologies in order to meet the demand for reduced size and high accuracy. A miniature system provides portability, low material and energy consumption, and a better integration and process automation [7]. To obtain a better precision, position control of a machine tool has become the primary concern. For precise positioning measurement, different techniques have been proposed, among them the use of position sensing detectors (PSD), which are low cost and have high resolution [19].

### 2.3. Signal Processing Methodologies

In order to test the functionality of the proposed smart sensor network, four methods recently used in machine tools have been identified, where the required processing can be implemented in each sensor node due to its reconfigurability.

#### 2.3.1. Accelerometer-Based Vibration Monitoring

Rangel-Magdaleno *et al.* [29] use as part of their methodology an accelerometer-based vibration measurement for condition monitoring and failure detection utilizing the DWT (discrete wavelet transform) for detecting broken bars in induction motors. The DWT is computed using a set of discrete-time low and high-pass filters, which is known as the Mallat algorithm. The Mallat algorithm has two sections: decomposition (DWT) and reconstruction (IDWT). During the decomposition processes, the original signal goes through a low-pass and high-pass filter bank to obtain the low frequency components known as approximations, and the high frequency component as details. Each level of decomposition decimates the original data length to the half of the data in the previous level. According to the filter bank properties, the frequency band for an approximation *AC_L_* and a detail *DC_L_* is given by Equations (1) and (2) respectively, where *L* is the level of the desired decomposition and *fs* is the sampling frequency. The reconstruction section takes the information obtained from one or more decomposition levels and returns the signal representation to the time domain:

(1) $${\mathrm{AC}}_{L}=\left[0,\frac{\mathrm{fs}}{2^{L+1}}\right]$$

(2) $${\mathrm{DC}}_{L}=\left[\frac{\mathrm{fs}}{2^{L+1}},\frac{\mathrm{fs}}{2^{L}}\right]$$

#### 2.3.2. Jerk Monitoring

For jerk monitoring, Morales-Velazquez *et al*. [17] propose an antisymmetric high-order FIR filter based on a sensorless method for an encoder signal. To obtain the jerk signal from the position measurement, successive differentiations are required. A high order antisymmetric FIR filter is defined as a two-impulse window filter (TIWF) given by Equation (3), where filter order (*n*) is fixed and the impulse position (δ) depends on the adaptation method and *fs* is the sampling frequency:

(3) $$y(k)=\frac{f_{s}}{n-2\delta }\left\{-x(k-\delta )+x\left[k-\left(n-\delta \right)\right]\right\}$$

The position signal (*p*) from the encoder is prefiltered by a low-pass filter; then the signal speed (*ŝ*), acceleration (*â*) and jerk (*ĵ*) are estimated by cascading three TIWF to the filtered signal. To adjust the three TIWF, the jerk variance (*σ_j_^2^*) is calculated using (5), then, an adaptive function is applied and limited to obtain the adaptability parameter (*δ*) which defines the position of the impulse in Equation (3):

(4) $$\delta =4.4|{\text{log}}_{2}\left(\sigma_{j}^{2}\right)|$$

(5) $$\sigma_{j}^{2}=\frac{2}{n}\sum_{i=1}^{n/2}{\left(u_{i}-\bar{u}\right)}^{2}$$

where *u_i_* is the *i*-th jerk sample, and *ū* is the jerk mean in the interval [*i, n*/2*+i*].

#### 2.3.3. Tool Wear Monitoring

For tool-wear monitoring Trejo-Hernandez *et al*. [30] propose as part of their methodology an HSP-unit to determinate the flank-wear area using the signal current measurement. The feed motor current *I*, is the input signal to the current DAS (Data Acquisition System) driver. Then the current signal is time windowed to only take into account the time when the cutting is made during the machining process. The time windowing initializes its processing when the CNC machine indicates it through a logical signal *MS* (Machining Start). The current signal is processed with a low-pass 32-order FIR filter (cut-off frequency *f_c_* = 120 Hz). Afterwards, the *I_rms_* value of the current signal is obtained according to Equation (6), where *I_i_* represent the *i*-th sample and *m* the length of the windowed samples:

(6) $$I_{\mathrm{rms}}=\sqrt{\frac{\sum_{i=1}^{m}{\left(I_{i}\right)}^{2}}{m}}$$

In a next step, the flank-wear area *A_f_* is obtained by a polynomial approximation. The polynomial approximation is shown in Equation (7):

(7) $$A_{f}=\left(I_{\mathrm{rms}}\right)=213.8785I_{\mathrm{rms}}^{3}-175.1362I_{\mathrm{rms}}^{2}+47.5507I_{\mathrm{rms}}-4.1563$$

#### 2.3.4. PSD-Based Micropositioning Measurement

A PSD is a device in a substrate of photodiodes divided into two or four parts capable of measuring position from a light beam regardless of the shape of the beam and the intensity distribution [19]. Owing to its resolution, this device has been used for micropositioning measurements in systems for micro-machining applications.

There are different sensors for micro-displacement measurement, which integrate a LED emitter along with a PSD sensor in the receptor where the output signal is a proportional voltage to the distance. Because of the light environmental conditions or other factors, the output signal requires a statistic process like RMS calculation during the time of beam emission in order to obtain the distance signal. The *V_rms_* value of the voltage signal is obtained according to Equation (8), where *Vi* represents the *i*-th sample and *m* is the number of samples for each calculation:

(8) $$V_{\mathrm{rms}}=\sqrt{\frac{\sum_{i=1}^{m}{\left(V_{i}\right)}^{2}}{m}}$$

Sze-Wei *et al.* [19] proposed the use of a PSD sensor along with a servosystem based on a piezoelectric actuator in order to measure the translational error from a high precision stage. The servo system is controlled for the movement while the position error is measured with the PSD. The profile of the measured error by the PSD was stored during machining for servosystem compensation.

#### 2.3.5. Characteristics of the Sensors

The characteristics of primary sensors to be used in the methodologies depend on the application needs. For instance, in the case of accelerometer-based vibration monitoring, the resolution of the sensor is determined depending on the level of detail needed and the accuracy depends on the vibration measurement range in the application; however, the methodology is suitable for any type of accelerometer. For jerk estimation, encoder resolution (counts per revolution) does not affect the algorithm of the methodology proposed, however a higher accuracy of the sensor will provide a better speed control. In the case of tool wear monitoring, a higher resolution will provide a better estimation of the flank wear area. Micropositioning methodology works for any sensor resolution but this will limit the minimum compensation of error and therefore the accuracy of the positioning system. Finally, each methodology can be adapted for the required resolutions, either by the sensor or by the number of bits at the analog to digital converter which require a change in data format.

## 3. Wireless Smart Sensor Network

**Figure 1.**
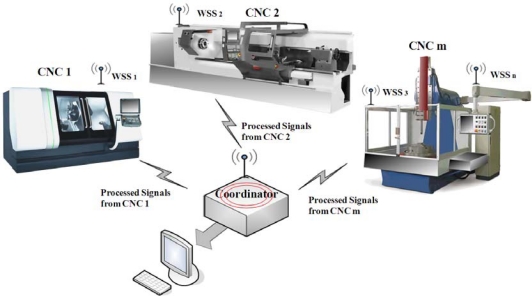
General block diagram of the smart sensor network.

Figure 1 shows the general block diagram of the network, which can integrate *n* wireless smart sensors (WSS) based on the proposed platform, the maximum value of *n* is 65,535 for a single coordinator. The smart sensors are connected to different primary sensors that monitor the variables of interest from the CNC machine in a typical manufacturing cell. The master device or coordinator is implemented in a platform with similar characteristics, it is responsible for associating or disassociating the devices in its PAN, and it configures the processing and acquisition parameters. The network is capable of communicating directly with a computer, a display or a storage system.

**Figure 2.**
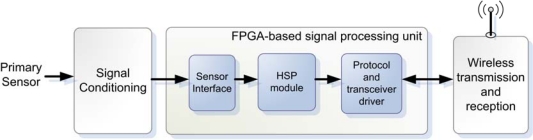
Wireless smart sensor system stages.

The implemented platform consists of three stages as shown in Figure 2: signal conditioning, wireless transmission and reception, and hardware processing and control. The signal conditioning stage includes the instrumentation of the different type of sensors that are incorporated on the device; this stage is developed in an external module with a physical interface for coupling to the platform. This module is different for each type of sensor, and its construction is simple and compact. The wireless transmission and reception stage is constituted by a module that allows the modulation and transmission of data; this stage includes a commercial transceiver circuit, which uses the standard IEEE 802.15.4 in the physical and media access control (MAC) layer. Furthermore, it includes the physical environment for wireless transmission, composed of coupling circuitry for an external antenna.

The hardware processing unit is based on a low-cost and low-power consumption FPGA that controls the interface of the signal conditioning stage by implementing the driver for the analog-to-digital converter (ADC), which depends on the primary sensor connected to the platform. It connects to the transmission stage in order to control the radio transceiver for the wireless communication; it also handles the communication protocol according to the network capabilities and includes modules for hardware signal processing.

### 3.1. Wireless Protocol

In the hardware processing unit it is necessary to define a communication protocol for the wireless sensor network. Since the selected standard is capable of operating at three different radio frequency bands, the 2.4 GHz band is chosen because of its high transmission rate and because it operates in the industrial, scientific and medical (ISM) band.

The communication protocol uses a star topology network in which slave nodes can communicate only with the coordinator of the network [28]; each node has a specific 16-bit PAN address, and at the same time a 16-bit and 64-bit individual addresses all stored in a memory into the device. The network coordinator integrates several tasks as: association and disassociation of nodes in the network, device configuration, data request and data reception, request of node status. Moreover, the nodes integrate specific tasks for the sensor network: association to only one coordinator, send data to request and receive setup instructions; that is why they are called reduced functionality devices (RFD) as they have basic functionalities in the protocol but they are not capable of coordinating a network of similar devices.

**Figure 3.**
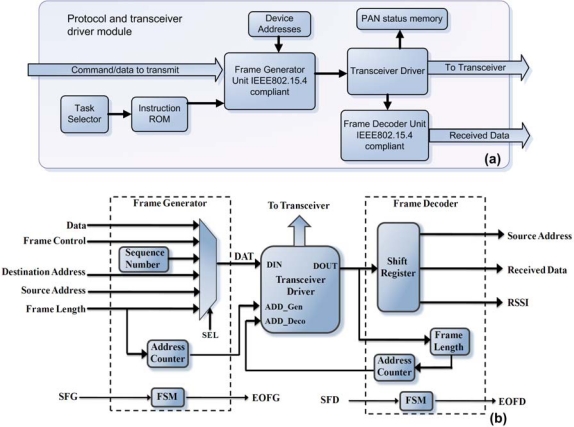
Block diagram of **(a)** the protocol architecture, **(b)** frame-generator and frame-decoder modules detail.

Figure 3a shows the implemented architecture to manage the wireless transmission stage; it consists of two parts: the first part is an interface to the transceiver circuit for physical configuration of the device; it configures the MAC-layer characteristics and manages the transmission and reception options. The second part includes the implementations of the proprietary protocol that is based on an instruction read-only memory (ROM) that stores the parameters for the physical initialization of the transceiver; a frame-generator module and a frame-decoder module are implemented in this part according to the frame format required by the standard.

Memory in the transceiver is implemented as static RAM and it is accessible via the SPI port. Memory is functionally divided into control registers and data buffers (FIFOs), to handle the reception and transmission of packets. The frame-generator unit is shown in Figure 3b; it contains a field selector and an address counter to write through the transceiver driver the sequence of data in the transmission FIFO (TXFIFO) according to the frame format specified by the standard containing several fields; frame length, frame control, sequence number, PAN identifier and addressing fields and the data payload that contains the information from the HSP-modules. The first field is the frame length calculated depending of the number of data to be sent; the second field is the frame control defined by the standard and contains information of the frame structure. An 8-bit counter is used to generate the sequence number, this counter is incremented after the previous transmission is succeeded, and PAN identifier and addressing fields are located in a memory outside the module in order to be reconfigured. This module has an FSM (finite-state machine) to control the selector and the counters. The unit has a start frame generation (SFG) and end of frame generation (EFG) ports, in order to be externally controlled.

The frame-decoder unit, shown in Figure 3b, is constituted by a shift register, a frame length counter, an address counter and an FSM, once the transceiver has received a valid packet without errors an interrupt occurred and the packet is read, the first byte read from the reception FIFO (RXFIFO) is the frame length and the data is stored in a register in order to read only the packet, after the frame length is stored the following data is read and stored in the shift register, the fields are organized according to their origin and importance, extracting only the source address and the data payload. Each packet has information of the signal received; the received signal strength indicator (RSSI) can be extracted from the packet too. Also this module has a start frame decoder SFD port and end of frame decoder EOFD port to control the module externally.

A memory bank is defined with the structure of the instruction ROM that defines the tasks of this protocol. Within the instruction ROM, there is a section that contains the initialization parameters of the different devices, defines the type of network, the frame format, the transmission power, and the communication channel. The coordinator module keeps all its addresses fixed, while the node addresses are defined by the coordinator.

### 3.2. Reconfigurable Processor

**Figure 4.**
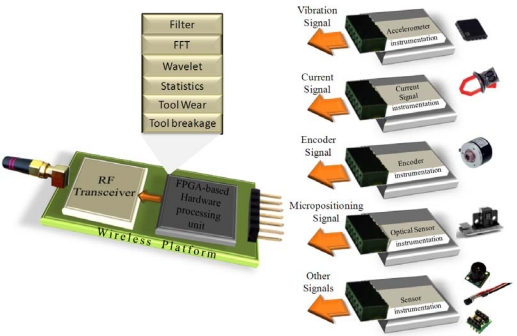
Block diagram of the wireless platform.

The main feature of the platform lies upon its capacity of incorporating different HSP modules; these modules can be implemented in the hardware processing stage due to its reconfigurability. Figure 4 shows a block diagram of the proposed platform overall structure that depicts the FPGA-based processing stage where the description of an interface is implemented; this interface depends on the specific sensor connected to the platform, and on the drivers for the wireless module. Figure 4 also shows the wireless transmission stage that consists of a radio frequency transceiver and the corresponding coupling circuitry. According to the application, different signal conditioning systems can be connected to the wireless platform depending on the sensors required for such an application.

#### 3.2.1. Vibration Monitoring HSP Core

**Figure 5.**
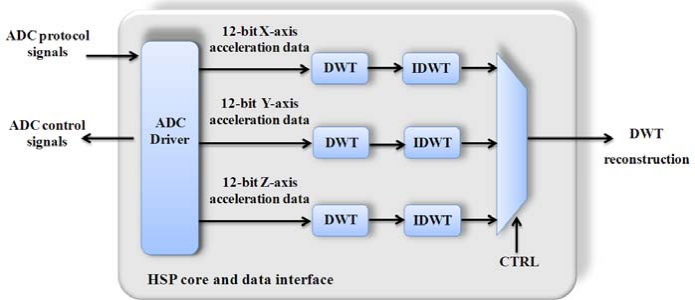
Block diagram for vibration monitoring utilizing the HSP core.

For broken-bar detection in spindle induction motors of CNC machines, the implementation shown in Figure 5 is used. According to the methodology proposed by Rangel-Magdaleno *et al*. [29], a module that manages the ADC converter, and the modules of the wavelet transform for the decomposition (DWT) and the reconstruction (IDWT) of the selected level of analysis, were included. Once the reconstruction for each axis is obtained during the startup transient, the axis that will be sent through the wireless platform is selected.

#### 3.2.2. Jerk Monitoring HSP Core

**Figure 6.**
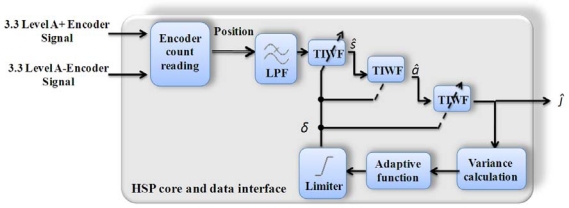
Block diagram for jerk monitoring utilizing the HSP core.

Figure 6 shows the block diagram of the implemented methodology for jerk monitoring proposed by Morales-Velazquez *et al*. [17]. The implemented algorithm includes modules to acquire the position signal from an encoder, a low-pass filter (LPF) and TIWF filters to estimate velocity (*ŝ*), acceleration (*â*) and jerk (*ĵ*), a module for variance calculation, and a module for obtaining the adaptability parameter (*δ*).

#### 3.2.3. Tool Wear Monitoring HSP Core

**Figure 7.**
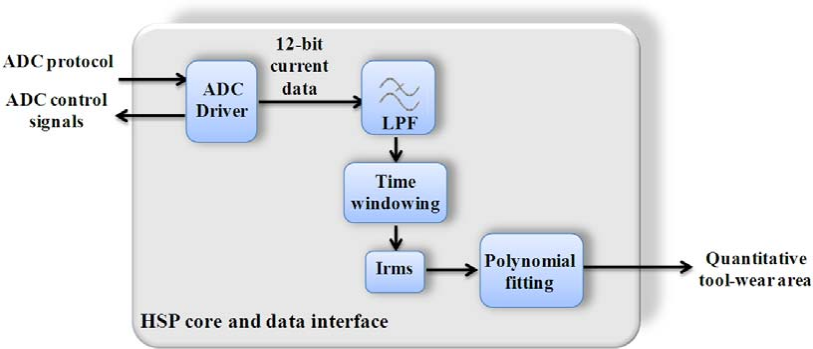
Block diagram for tool wear monitoring utilizing the HSP core.

For tool wear monitoring, the implementation was made as shown in the block diagram of Figure 7, and it was based on the case of study for current signal in the work of Trejo-Hernandez *et al*. [30]. To estimate the tool wear area from the spindle-driver current measurement, the implemented algorithm includes a driver for an ADC converter, a low pass filter (LPF), a time windowing for taking the signal during machining process only, a module for computing the RMS value of the current signal, and a polynomial approximation for calculating quantitatively the tool-wear area as shown in (7).

#### 3.2.4. PSD-Based Micropositioning Measurement HSP Core

**Figure 8.**
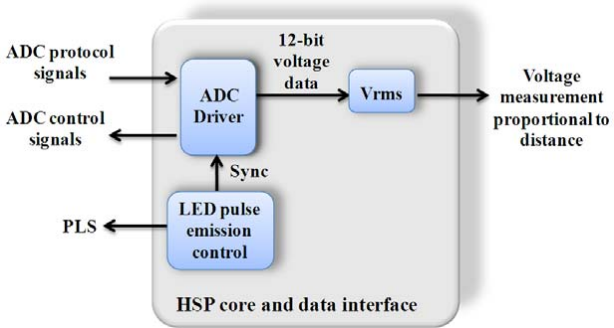
Block diagram for micropositioning measurement utilizing the HSP core.

Figure 8 shows the block diagram for micropositioning measurement based on a PSD sensor. The implementation consists of a driver for the ADC, a module for the LED pulse emission control (PLS) and an RMS computation unit for obtain the voltage measurement proportional to the distance.

### 3.3. Instrumentation

For measuring the corresponding variables according to the application, signal conditioning from the primary sensors is necessary; these signals are adapted to the wireless platform through a board-to-board connector. This section presents the signal conditioning implementation for each sensor used in the corresponding methodology.

#### 3.3.1. Acceleration Sensor Board

**Figure 9.**
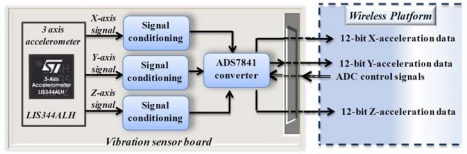
Block diagram for 3-Axis Accelerometer instrumentation.

Figure 9 shows the block diagram of the instrumentation system utilized for measuring vibrations. This system consists of a triaxial accelerometer LIS344ALH from STMicroelectronics [31], which has a bandwidth of 1,500 Hz for all axes, a user-selectable full scale of ±2 g/±6 g (*g* = 9.81 m/s^2^), a 0.66 V/g sensitivity and a 5 × 10^−4^ resolution over a 100 Hz bandwidth. The system also contains a 12-bit resolution at the 4-channel ADC from Texas Instruments ADS7841 [32], with a maximum sampling rate of 200 KHz for the four channels, the unused channel is left unconnected for this application. The control signals and output of the ADC are connected to the interface with the wireless platform.

#### 3.3.2. Current Sensor Board

**Figure 10.**
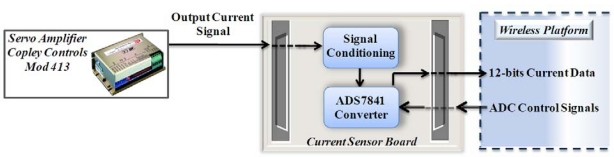
Block diagram for current signal instrumentation.

Figure 10 shows a block diagram of the instrumentation system for the feed motor current measurement that is obtained from a typical Copley Controls Corp servoamplifier model 413 with a 5 V/A sensitivity. The resolution of the system is 12-bit given by a 4-channel ADC ADS7841, also a instrumentation for signal conditioning of the motor current is required. This system can measure simultaneously up to four current signals; for this case study, only one channel is used, leaving the others connected to ground.

#### 3.3.3. Encoder Sensor Board

**Figure 11.**
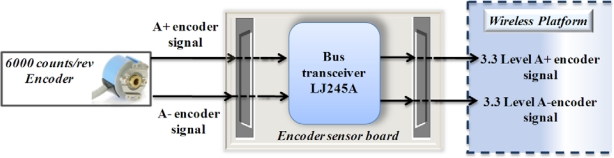
Block diagram encoder signal instrumentation.

Figure 11 shows the block diagram of the instrumentation system for an encoder with a resolution of 6,000 counts per revolution that is coupled to the servomotor on one axis of a CNC machine. For conditioning the encoder signals, a LJ245A driver is used in order to make the voltages levels from the encoder compatible with the platform.

#### 3.3.4. Position Sensor Board

**Figure 12.**
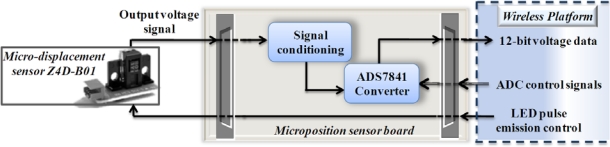
Block diagram for micro-displacement sensor instrumentation.

The block diagram for micro-displacement measurement is shown in Figure 12; the instrumentation system is made for an optical sensor Z4D_B01 based on a PSD with a resolution of 10 μm and a 1.4 mv/μm sensitivity. The system contains a 12-bit resolution, 4-channel ADC ADS7841 to convert the voltage signal from the sensor. The instrumentation only uses one channel and the others are disabled as recommended by the manufacturer. A control signal is sent from the wireless platform to control the emission pulse of the infrared LED in the sensor.

## 4. Experimental Section

In this section the experimental validation of the sensor network applied to a manufacturing cell is presented. The experimentation includes three CNC machines. Four methodologies are implemented in four identical wireless platforms. The test consists on monitoring the smart wireless sensor during the operation of the CNC machines simultaneously.

**Figure 13.**
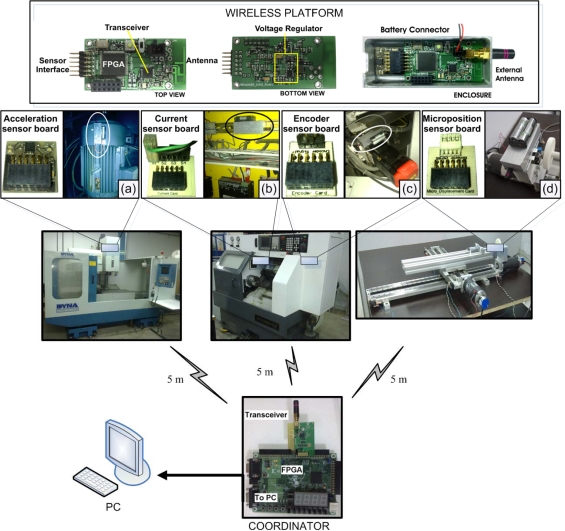
Experimental setup in a manufacturing cell, **(a)** vibration WSS mounting on the motor of DYNA-4M, **(b)** current WSS mounting on the FANUC Oi Mate-TC lathe panel, **(c)** encoder WSS mounting on a motor of FANUC Oi Mate-TC lathe, **(d)** micropositioning WSS mounting on a two-axis linear stage.

The experimental setup consists on assembling four wireless smart sensors on three CNC machines within a manufacturing cell. Each smart sensor uses an AGLN250ZV2 low cost and low power consumption FPGA as processing unit. They operate at 20 MHz and have a radio frequency commercial transceiver MRF24J40 that is compatible with the IEEE 802.15.4 standard. Figure 13 shows one of the four developed wireless smart sensor nodes with its corresponding enclosure for mounting and also shows the smart sensor placements in the manufacturing cell, the network coordinator is located in an equidistant space from the nodes and it is implemented in a commercial FPGA platform Spartan-3 XC3S200 that is connected to a PC, which uses the transceiver module MRF 24J40 too.

In a CNC machine center, a WSS is mounted on the spindle of the principal motor of a DYNA-4M CNC system to measure and analyze vibrations, and to detect broken bars in the induction motor as shown in Figure 13a. Two conditions are set in the induction motor: the first one is for a healthy motor and the second one is for a motor with two broken bars. The acquisition is performed at a rate of 1,500 samples per second. In a machining process of a conic piece, the quantitative estimation of the tool wear area is obtained for three CNMG433MA inserts with different flank-wear area. Figure 13b shows the mounting of a WSS on a FANUC Oi Mate-TC lathe; the feed rate is 0.333 mm/rev, the cutting depth is 1.5 mm and the cutting speed is 72 m/min. During the same machining process, the encoder position is measured on the X-axis to estimate the jerk at 1,000 samples per second, the WSS mounting is shown in Figure 13c. On the other hand, for micropositioning measurement, a movement profile in micrometers is defined for a precision stage coupled to an EZG705-0-101 servomotor; the mounting of the WSS for this case is shown in Figure 13d. Table 2 shows the FPGA resource utilization for the synthesis of each smart sensor corresponding to each of the methodologies implemented.

Table 3 shows the FPGA resource utilization for the synthesis of the coordinator module. This resource utilization is the same independently of the nodes integrated in the network, the maximum power consumption of coordinator is 68.99 mW.

## 5. Results and Discussion

In this section the results of the techniques used in each smart sensor are presented, the information obtained simultaneously in the wireless sensor network is stored on the coordinator RAM and then, it is transferred to a PC, finally, using Matlab, the results are plotted.

### 5.1. Vibration Results

In this experiment, the motor of the spindle with two broken bars was used in order to test the performance of the network; a latter test was carried out for a healthy motor under the same network conditions.

**Figure 14.**
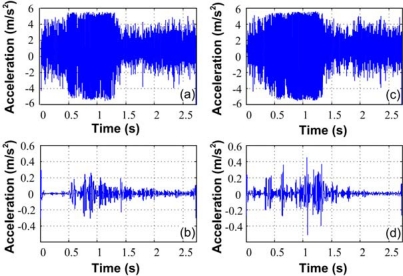
Vibration Signals for induction motor, **(a)** time-domain signal for healthy motor, **(b)** DWT reconstruction level 5 for a healthy motor, **(c)** time-domain signal for a motor with two broken bars, **(d)** DWT reconstruction level 5 for a motor with two broken bars.

Figure 14a and Figure 14c show the time-domain vibration signal from the healthy motor and a motor with two broken bars respectively; the DWT analysis at a level-5 decomposition and reconstruction for a healthy motor is shown in Figure 14b and for a motor with two broken bars is shown in Figure 14d. Both signals are transmitted in this test for comparison.

### 5.2. Current Results

**Figure 15.**
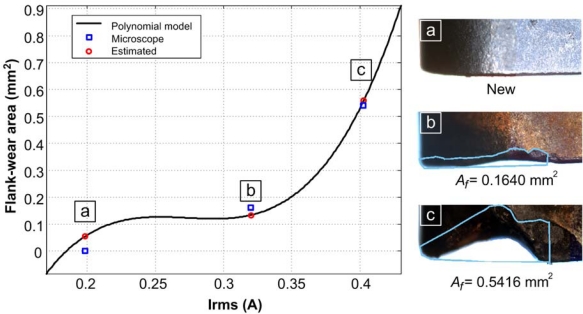
Flank-wear area estimation showing the micrograph of selected inserts and their corresponding tool-wear area.

The results from the current technique are shown in Table 4. The flank-wear area is estimated in each machining process. The quantitative value of the area is transmitted from the WSS in a 2.22 fixed point format after the machining process. Figure 15 shows the cutting tool micrograph and the estimation data based on the polynomial function of Equation (7) for the quantitative flank-wear area for selected inserts

### 5.3. Encoder Results

**Figure 16.**
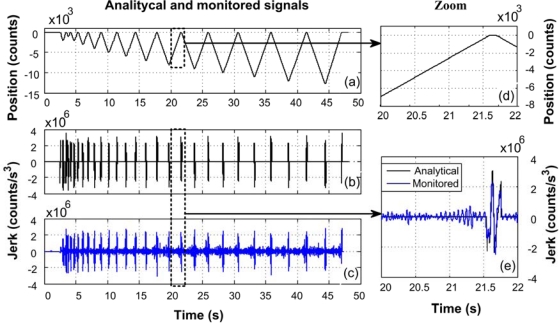
**(a)** Position, **(b)** analytical jerk, **(c)** monitored jerk, **(d)** zoom of position, **(e)** jerk analytical and estimated.

Figure 16 shows the results obtained from the encoder reading on the lathe during the machining process. Figure 16a shows the position sent by the WSS in order to obtain analytically the jerk as shown in Figure 16b, and the comparison against the WSS estimation shown in Figure 16c.

### 5.4. PSD Results

**Figure 17.**
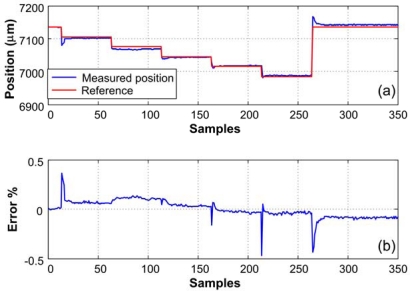
**(a)** Reference and measured position, **(b)** error of the measured position.

The voltage measurement proportional to distance delivered by the sensor is sent through a WSS to a PC; the distance is obtained graphically in the order of micrometers; then, it is compared to the sent position as shown in Figure 17a. Finally, the error is calculated and shown in Figure 17b.

### 5.5. Speed and Range Network Results

**Figure 18.**
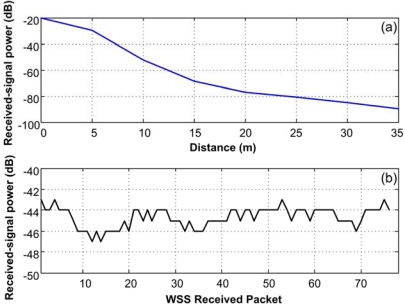
Received-signal power at different distances.

Figure 18a shows the signal strength variation of a node for various distances; Figure 18b shows the signal strength for the vibration WSS during the test.

Table 5 summarizes the network utilization for each smart sensor; the bits per second that are read directly from the sensors, and the amount of information after signal processing, which was 42,365 bps.

### 5.5. Network Capabilities

To determine the maximum number of WSS that can be integrated in the network, it is necessary to determine the maximum smart-sensor data rate that can be sent through the channel, which depends on the HSP-core implemented in the nodes and the maximum data rate that the coordinator can receive. The coordinator can read a complete frame with the maximum data payload in 263.64 μs; however, the standard specifies a maximum data rate of 250 kbps. If the network integrates nodes with low data rates, the number of nodes increase, for example a network with nodes transmitting at 1 byte per second with the minimum frame format, the netwok can integrate up to 2,604 nodes, but if the node transmit information at 232,283 bps all the bandwith is used and only one node can be integrated in this network with good conditions of reception.

### 5.6. Discussion

Experimental results using the proposed wireless smart sensor network validate the four methodologies implemented for CNC machines. The first methodology based on accelerometer measurement uses a smart sensor for detecting broken bars in spindle induction motors by using a DWT transform. The second methodology measures the servomotor driver current and the processing implemented on the smart sensor to estimate the flank-wear area in inserts during machining. For the third methodology, the validation was done by using a smart sensor for the measurement of the encoder signal from an axis servomotor of a CNC machine. The measure of position for a linear stage was obtained by processing in a smart sensor the voltage signal obtained from an optical sensor, calculating RMS value. For this experiment the PAN coordinator was placed at 5 m from the sensor nodes, allowing an optimum data reception and a bandwith network utilization of 22.53% of the total available of an IEEE 802.15.4 channel. During the test, additional data was transmitted in order to show the results validation, as in the case of accelerometer-based vibrations monitoring and encoder-based jerk monitoring methodologies; the omission of this additional information reduces the traffic in the network. The amount of data sent through is considerably reduced from 660,960 bps to 42,365 bps, 6.4% of the total raw data read. The applied processing allows the relevant information to be sent through the network for each particular application; it is also clear that the raw-data rate is over the maximum transfer rate in the specifications of the used standard [28]. By using HSP modules in the WSS, the transmission time of the smart-sensor data after processing is significantly reduced in comparison with the raw data from the sensors; in this way, decreasing the traffic through the communication channel reduces the overall processing time, and increases the number of WSS that can be handled simultaneously in the network.

## 6. Conclusions

The wireless smart sensor network developed in this work is capable of coordinating different nodes for the measurement and processing of several variables in the new generation of machine tools under a completely hardware reconfigurable platform. The wireless communication based on IEEE 802.15.4 standard allowed fulfillment of the requirements for new generation CNC machines analysis and monitoring in a manufacturing cell, with the capability to incorporate a large number of variables for measuring and procesing. The proposed reconfigurable smart sensors can perform complex processing, which is optimal in a sensor network for condition monitoring and data analysis in a machining center, since each smart sensor pre-processes the information, reducing the amount of data to be sent through the network. The outcome of this capability provides a more efficient decision-making in the manufacturing cell, contrary to the supposed fact of performing a post-processing for all raw data obtained from the measurement of each variable. The application of the smart sensor network developed in this work can be suitable for monitoring and processing of other variables and other process where on-line measurement is required, as in a production line.

## Figures and Tables

**Table 1.** Comparative between WiFi, Bluetooth and ZigBee standards.

**Standard**	**IEEE 802.11g (WiFi)**	**IEEE 802.15.1 (Bluetooth)**	**IEEE 802.15.4 (ZigBee)**

**Bandwidth**	22 MHz	1 MHz	2 MHz
**Power consumption**	400 mA–20 mA	40 mA–0.2 mA	30 mA–3 μA
**Transmission rate**	11 Mbps–54 Mbps	1 Mbps	250 kbps
**Number of devices**	32	7	65536
**Latency**	Enumeration up to 3 s	Enumeration up to 10 s	Enumeration up to 30 ms
**Transmission range**	100 m	10 m	100–1,000 m
**Security**	SSID	64 bits,128 bits	128 bits, AES
**Available physical channels**	3	4	16

**Table 2.** FPGA resources utilization for each smart sensor.

**Smart sensor**	**Cells Used/Available**	**Cells Used Percentage %**	**Block RAMs Used/Available**	**Block RAMs Used Percentage %**	**Power consumption (mW)**

Current	5947/6144	97	1/8	12	9.676
Encoder	4989/6144	81	5/8	62	12.236
Vibration	6081/6144	98	8/8	100	16.219
Micropositioning	2553/6144	42	0/8	0	7.359

**Table 3.** FPGA resources utilization for the coordinator *.

Element	Used	Available	Percentage %
Slice Flip Flops	402	3840	10
4 input LUTs	1039	3840	27
Slices	585	1920	30
Block RAMs	0	12	0
Multipliers	0	12	0

* 200,000-gate Xilinx Spartan-3 FPGA: XC3S200

**Table 4.** Flank-wear area results by current and tool micrograph.

**Measure**	**Current (A)**	**Flank-wear area estimation (mm^2^)**	**Micrograph flank-wear area (mm^2^)**

1	0.19833	0.05401	0
2	0.33059	0.15035	0.1640
3	0.40166	0.54750	0.5416

**Table 5.** Network utilization for raw data and processed data transmission.

**Technique**	**Sampling rate (Samples/s)**	**Raw data format (bits)**	**Raw data (bps)**	**Output rate (Data/s)**	**Smart sensor data format (bits)**	**Smart sensor data (bps)**

Current	1,500	12	18,000	0.2	24	24
Encoder	1,000	24	24,000	1,000	24	24,000
Vibration	1,500	12	54,000	1,500	12	18,000
Micropositioning	47,080	12	564,960	20	18	360
